# Irrigation in a Recent Wound

**Published:** 1875-05-15

**Authors:** Charles Hervey Hunt

**Affiliations:** Stanwood, Iowa


### MEDICAL EXAMINER.

A

*Semi-Monthly Journal of Medical Sciences.*

EDITED BY N. S. DAVIS, M.D., AND F. H. DAVIS, M.D.

No. X. CHICAGO, MAY 15, 1875. VOL. XVI.

### Original Communications.

### IRRIGATION IN A RECENT WOUND.

By CHARLES HERVEY HUNT, M.D., STANWOOD, IOWA.

CHARLES A. P., who was yard-master of the railroad at this place, while attempting to jump upon the pilot or cowcatcher of an engine, had the misfortune to slip, bringing his foot between the pilot and the rail. The space in which the foot was caught, was afterward ascertained by running the engine over the same place, and was found to be about three-fourths of an inch. The foot, upon examination, presented the appearance of a foot that had just come from under a trip-hammer. The bottom of the foot was burst open from the ball of the heel to the great toe, so that all the metatarsal bones, and the tendons, could be seen. The ball of flesh under the first toe was split off. Branching off from the main rent, was one that extended up between the fourth and little toe to the top of the foot, then across to the inside of the foot.

Fortunately, no bones were broken, except a piece from the metatarsal of the small toe at its articulation with the phalanx. The patient was put under the influence of an anæsthetic, and the wound was cleansed of blood and dirt, and dressed in the regular manner. As soon as the effects of the anæsthetic were gone, the pain returned with great severity. Anodynes did not seem to have any effect. At this point, the use of cold water occurred to me. A large tub was brought in with a pail of water drawn from the bottom of the well. The bandages were nearly all removed, leaving the adhesive straps so that the water could come in contact with the flesh. A thin cloth was laid over the foot, and upon this the water was poured in a stream, about the size of a pipe-stem. Within a few moments the pain began to abate, and in twenty minutes the patient was resting easy. This was kept up all night, and on my visit in the morning, the pouring of the water was still going on. Whenever it was stopped for a few moments, the pain returned, and the relief obtained by the use of the water was so great, that the patient would not allow it to be stopped; and agreeable to his wish, it was kept running night and day for one week, when its use was discontinued, as the pain had nearly ceased.

There was no sloughing, and the discharge was small. The swelling was not more than one-third as much as there would be in such a wound where other means are used, and it did not extend above the ankle. After the use of the water was discontinued, the ordinary dressing of carbolized oil was used. The recovery was rapid, and the foot, with the exception of a partial stiffness in the first toe, is as good as ever. I have found but little said in our text books on surgery, upon the use of water in wounds, fractures, dislocations or sprains. In these cases, it seems to me that the great object to be obtained is, the prevention of the congestion of the parts, which is followed by swelling and exudation, and I know of nothing that will do it more effectually than cold water applied continuously. A little ingenuity on the part of the physician, will be all that is necessary for the making and arranging of apparatus for holding and conveying water to the part. I believe with the professor who says, "give us plenty of fresh air," but give us also, plenty of cold water.

